# Generation and characterization of interferon-lambda 1-resistant H1N1 influenza A viruses

**DOI:** 10.1371/journal.pone.0181999

**Published:** 2017-07-27

**Authors:** Natalia A. Ilyushina, Vladimir Y. Lugovtsev, Anastasia P. Samsonova, Faruk G. Sheikh, Nicolai V. Bovin, Raymond P. Donnelly

**Affiliations:** 1 Division of Biotechnology Research and Review II, Center for Drug Evaluation and Research, U.S. Food and Drug Administration, Silver Spring, Maryland, United States of America; 2 Division of Viral Products, Center for Biologics Evaluation and Research, Food and Drug Administration, Silver Spring, Maryland, United States of America; 3 Carbohydrate Chemistry Laboratory, Shemyakin Institute of Bioorganic Chemistry, Russian Academy of Sciences, Moscow, Russia; University of South Dakota, UNITED STATES

## Abstract

Influenza A viruses pose a constant potential threat to human health. In view of the innate antiviral activity of interferons (IFNs) and their potential use as anti-influenza agents, it is important to know whether viral resistance to these antiviral proteins can arise. To examine the likelihood of emergence of IFN-λ1-resistant H1N1 variants, we serially passaged the A/California/04/09 (H1N1) strain in a human lung epithelial cell line (Calu-3) in the presence of increasing concentrations of recombinant IFN-λ1 protein. To monitor changes associated with adaptation of this virus to growth in Calu-3 cells, we also passaged the wild-type virus in the absence of IFN-λ1. Under IFN-λ1 selective pressure, the parental virus developed two neuraminidase (NA) mutations, S79L and K331N, which significantly reduced NA enzyme activity (↓1.4-fold) and sensitivity to IFN-λ1 (↓˃20-fold), respectively. These changes were not associated with a reduction in viral replication levels. Mutants carrying either K331N alone or S79L and K331N together induced weaker phosphorylation of IFN regulatory factor 3 (IRF3), and, as a consequence, much lower expression of the IFN genes (*IFNB1*, *IFNL1* and *IFNL2/3*) and proteins (IFN-λ1 and IFN-λ2/3). The lower levels of IFN expression correlated with weaker induction of tyrosine-phosphorylated STAT1 and reduced RIG-I protein levels. Our findings demonstrate that influenza viruses can develop increased resistance to the antiviral activity of type III interferons.

## Introduction

Influenza A viruses are respiratory tract pathogens that can cause widespread annual epidemics and occasional pandemics with a huge impact on public health [1]. Recent sporadic human infections with H5N1 and H7N9 avian influenza viruses have also raised concerns regarding the pandemic potential of these viruses [2–4]. Currently, two classes of FDA-approved drugs are recommended for both preventative use and treatment of infected patients: M2 ion channel inhibitors (amantadine, rimantadine) and neuraminidase (NA) inhibitors (oseltamivir, zanamivir) [5,6]. Additionally, several new antiviral agents have been approved in some countries, including two NA inhibitors (peramivir and laninamivir) [7–10] and a viral polymerase inhibitor (favipiravir (T-705)) [11–13]. However, a major problem regarding anti-influenza drugs that target viral proteins is the frequent emergence of drug-resistant viruses. Most human H1N1 and H3N2 strains, including pandemic 2009 H1N1, are now resistant to amantadine/rimantadine [14–17]. Moreover, oseltamivir-resistant H1N1 viruses emerged rapidly and spread worldwide between 2007 and 2009 [18–20]. Therefore, concern regarding the emergence of drug-resistant influenza viruses is clearly warranted. There is an urgent need for new antiviral agents to treat influenza virus infection while minimizing the potential for drug resistance.

Virus-induced interferons (IFNs) are a complex group of biologically active cytokines that include type I and type III IFNs. These cytokines represent a major component of the innate immune response to influenza virus infection [21–24]. Type I IFNs, including IFN-α and IFN-β, signal through a common cell surface receptor known as the IFNAR complex that is present on most cell types [21,22–25]. Type III IFNs include IFN-λ1, IFN-λ2, IFN-λ3, and IFN-λ4, and these proteins signal through a distinct heterodimeric membrane receptor composed of the IFN-λ-specific IFN-λR1 chain and the IL10R2 chain [26,27]. Expression of type III IFN receptors is largely restricted to cells of epithelial origin, including epidermal, pulmonary, and gastrointestinal epithelial cells [27–29]. Consequently, type III IFNs have a more limited functional range than type I IFNs, and are likely to mediate distinct functional roles from the type I IFNs *in vivo*.

Influenza virus infection induces co-expression of type I and type III IFNs by target cells such as airway epithelial cells [1]. The subsequent binding of type I and type III IFN proteins to their cognate receptors results in rapid activation of the JAK-STAT signaling pathway. This in turn leads to phosphorylation of signal transducer and activator of transcription 1 (STAT1) and STAT2 and formation of active IFN-stimulated gene factor 3 (ISGF3) complexes which then induce transcriptional activation of a large set of IFN-stimulated genes (ISGs). These ISGs encode a wide variety of antiviral proteins that function to restrict influenza virus replication [21,23,25–27].

Several recent studies have shown that both type I and type III IFNs can exert antiviral activity against influenza virus infection. For example, Osterlund et al. [30] demonstrated that both seasonal and 2009 pandemic H1N1 influenza A viruses are sensitive to the antiviral actions of type I (IFN-α/β) and type III (IFN-λ1 and -λ3) IFNs in human monocyte-derived dendritic cells and macrophages. Pretreatment with IFN-α/β or IFN-λ significantly inhibited the replication of influenza A and B viruses [31,32], including highly pathogenic H5N1 avian influenza viruses [28,29,33,34]. In a guinea pig model, intranasal administration of human recombinant IFN-α significantly reduced lung and nasal wash titers of a reconstructed 1918 pandemic H1N1 virus as well as a contemporary H5N1 strain [35]. Furthermore, a recent report from our group demonstrated that type I IFN (IFN-β) and type III IFN (IFN-λ1) used alone or in combination with oseltamivir carboxylate induced significant antiviral activity against influenza A viruses *in vitro* [36].

In light of the critical antiviral action of IFNs and their potential use as anti-influenza agents, it is important to understand whether resistance to these host proteins can develop. To date, no published studies have examined the potential emergence of IFN-λ1-resistant influenza viruses. To gain insight into the probability of emergence of IFN-λ1 resistance, we generated IFN-λ1-resistant mutants of the 2009 pandemic A/California/04/09 (H1N1) virus by serial passage in a human airway epithelial cell line under IFN-λ1 selective pressure. We characterized the acquired viral genomic mutations, growth potential, and levels of resistance to IFN-λ1 of the selected viral mutants. We identified a single K331N mutation in the NA protein that is associated with markedly increased resistance to IFN-λ1.

## Materials and methods

### Cells, viruses, and reagents

The Madin-Darby canine kidney (MDCK) cell line, human embryonic kidney cell line (293T), and the human lung epithelial cell line (Calu-3) were obtained from the American Type Culture Collection (Manassas, VA, USA) and maintained as described previously [36,37].

Human influenza A/California/04/09 (H1N1) virus was kindly provided by Dr. Robert G. Webster (St. Jude Children’s Research Hospital, Memphis, TN). Stock virus was prepared by one passage in the allantoic cavities of 10 day-old embryonated chicken eggs for 48 h at 37°C, and aliquots were stored at −70°C until use. For the reverse-genetics generation of recombinant viruses, eight plasmids carrying the eight gene segments of A/California/04/09 (CA/04) virus were also kindly provided by Dr. Robert G. Webster. Recombinant viruses were generated by DNA transfection of 293T cells [37], and the point nucleotide mutations were inserted into the NA and M genes of the wild-type virus using a QuickChange site-directed mutagenesis kit (Stratagene, La Jolla, CA, USA). Virus stocks were prepared by incubation in 10 day-old embryonated chicken eggs for 48 h at 37°C. The entire HA, NA, and matrix (M) genes were sequenced to verify the presence of the desired mutations and absence of additional mutations. All experimental work was performed in a biosafety level-2 (BSL-2) laboratory approved for use of these strains by the U.S. Department of Agriculture and the U.S. Centers for Disease Control and Prevention.

Human recombinant IFN-λ1 protein was obtained from R&D Systems, Inc. (Minneapolis, MN, USA), and diluted in cell culture medium consisting of RPMI-1640 medium plus 10% fetal bovine serum (FBS) (Hyclone, Logan, UT, USA).

### Infectivity of H1N1 influenza viruses

The infectivity of H1N1 viruses was determined by plaque assay [38]. Briefly, confluent cultures of MDCK cells were incubated at 37°C for 1 h with 10-fold serial dilutions of each virus. The cells were then washed and overlaid with minimal essential medium (MEM) containing 0.3% bovine serum albumin, 0.9% Bacto agar, and 1 μg/ml l-(tosylamido-2-phenyl)ethylchloromethylketone (TPCK)-treated trypsin. After 3 days of incubation at 37°C, the cells were stained with 0.1% crystal violet in 10% formaldehyde solution, and the number of plaque-forming units (PFU) per milliliter and plaque size of any 10 plaques were determined using a Finescale magnifying comparator.

### *In vitro* susceptibility assay

The antiviral activity of IFN-λ was determined by measuring the reduction of cell-associated virus yields using an enzyme-linked immunosorbent assay (ELISA) [39]. Confluent monolayers of Calu-3 cells in 96-well plates were pretreated for 24 h with IFN-λ1 (-λ2, or -λ3) protein at concentrations ranging from 0.01 to 1000 ng/ml. After pretreatment, the cells were infected with influenza virus at a multiplicity of infection (MOI) of 0.01 PFU/cell, and incubated for an additional 24 h at 37°C. Virus replication was determined by measuring viral nucleoprotein levels on the surface of infected cells. The percent inhibition of virus replication was calculated from the absorbance values measured at 490 nm on a microplate reader (Bio-Rad Laboratories, Hercules, CA, USA) after correction for the absorbance values of the control uninfected cells. The absorbance values of the control wells without IFN-λ were used to establish the maximal levels of virus replication for these assays. At least three or four independent assays were performed to determine the 50% effective concentration (EC_50_) values for each virus. These values are defined as the concentration of IFN-λ necessary to reduce the cell-associated virus yield by 50% relative to that of the untreated wells.

### Virus yield reduction assay

The extracellular virus yield reduction assay was performed as described previously in 24-well plates containing confluent Calu-3 cells [36]. The concentrations of IFN-λ1 ranged from 1 to 1000 ng/ml (the 50% cytotoxic concentration for IFN-λ1 is ˃ 1000 ng/ml [36]) and IFN-λ1 was added to the 24-well plates for 24 h. After pretreatment, the cells were overlaid with 2× drug-containing medium (100 μl/well), infected with influenza virus at a MOI of 0.1 PFU/cell, and incubated for 48 h at 37°C. Virus yields were determined as the number of PFU/ml in MDCK cells. The drug concentration that caused a 50% decrease in the PFU titer in comparison to control wells without drug was defined as EC_50_. The results of two independent experiments were averaged.

### Viral replication kinetics

To determine multistep growth curves for each virus, Calu-3 cells were infected with the H1N1 viruses at an MOI of 0.001 PFU/cell. After incubation for 1 h, the cells were washed and overlaid with MEM medium containing 0.3% bovine serum albumin and 1 μg/ml TPCK-treated trypsin. The supernatants were collected at 6, 24, 48, 72, and 96 h post-infection and stored at -70°C until titration.

### Virus sequence analysis

Viral RNAs were isolated from virus-containing cell culture fluid after passages in Calu-3 cells or after transfection by using RNeasy Minikits (Qiagen, Germantown, MD, USA). Samples were reverse transcribed and analyzed by PCR using universal primers specific for influenza gene segments as described previously [40]. Sequencing was performed by the Research Central Facility for Biotechnology Resources at the U.S. Food and Drug Administration, Silver Spring, MD. The DNA template was sequenced using rhodamine or dichlororhodamine (drhodamine) dye terminator cycle sequencing Ready Reaction Kits with AmpliTaq DNA Polymerase FS (PerkinElmer Applied Biosystems, Waltham, MA, USA) and synthetic oligonucleotides. All samples were analyzed in a Perkin-Elmer Applied Biosystems DNA sequencer (model 373 or 377). DNA sequences were completed and edited by using a Lasergene sequence analysis software package (DNASTAR).

To characterize the virus population *in vitro*, 10 individual virus clones from two selected H1N1 variants, CA/04^+IFN-λ1^ and CA/04^−IFN-λ1^, were picked after 30 passages in Calu-3 cells and their HA and NA genes were sequenced. Clonal analysis of the virus populations confirmed the dominance of the mutations determined by sequencing after plaque purification in MDCK cells and showed that 9 out of 10 clones picked from CA/04^+IFN-λ1^ contained the double mutation (S79L and K331N, N1 numbering used throughout the text) in their NA genes.

### Receptor-binding assay

The binding of H1N1 influenza viruses to fetuin (containing α2,3- and α2,6-linked sialyl receptors) was measured in a direct solid-phase assay using the immobilized virus and horseradish peroxidase (HRP)-conjugated fetuin as described previously [41]. The affinity of the individual viruses for synthetic 3′- and 6′-sialylglycopolymers (3′SL/N or 6′SL) was measured in a competitive binding assay based on the inhibition of binding to peroxidase-labeled fetuin [42]. The association constant (*K*_a_) values were determined by Scatchard plot analysis as the sialic acid (Neu5Ac) concentration at the *A*_max_/2 point, where *A*_max_ represents maximum absorbance.

### NA enzyme activity and kinetics

The NA activity of influenza H1N1 viruses was measured by a fluorescence-based assay using the fluorogenic substrate MUNANA (Sigma-Aldrich, St Louis, MO, USA), based on the method of Potier et al. [43] as described previously [44]. Briefly, all recombinant viruses were standardized to an equivalent NA protein content of 0.015 ng/μl as determined by protein gel electrophoresis using purified and concentrated viruses. This virus dilution was selected as a dilution that converted ≤15% MUNANA substrate to product during the reaction time in order to meet the requirements for steady-state kinetic analysis [44]. Virus dilutions were prepared in enzyme buffer [32.5 mM of 2-(N-morpholino) ethanesulfonic acid (MES), 4 mM of calcium chloride, pH 6.5] and added (100 μl/well) in duplicate to a flat-bottom 96-well opaque black plate (Corning, Tewksbury, MA, USA). After preincubation for 20–30 min at 37°C, the MUNANA substrate at various concentrations (separately pre-incubated for 20–30 min at 37°C) was added to all wells (50 μl/well). Immediately after adding the MUNANA substrate, the plate was transferred to a 37°C pre-warmed SpectraMAX Gemini XPS microplate reader (Molecular Devices, Sunnyvale, CA, USA) and fluorescence was measured every 60 s for 60 min at 37°C, using excitation and emission wavelengths of 360 nm and 460 nm, respectively. Enzymatic reactions were performed under conditions where signal-to-noise ratios were above 10 during more than 30 min of the reaction time. Time course data from each concentration of the MUNANA substrate were examined for linearity by linear regression analysis. Data with *R*^2^>0.99 were used for analysis. The kinetic parameters Michaelis-Menten constant (*K*_m_) and maximum velocity of substrate conversion (*V*_max_) of the NAs were calculated by fitting the data to the appropriate Michaelis-Menten equations by using nonlinear regression in Prism 6.0 software (GraphPad Software, La Jolla, CA, USA). Values are the means of three independent determinations.

### Minigenome assay for polymerase activity

Subconfluent monolayers of 293T cells (7.5 × 10^5^ cells in 35-mm dishes) were transfected with the luciferase reporter plasmid (enhanced green fluorescent protein [EGFP] open reading frame in pHW72-EGFP replaced with the firefly luciferase gene) [45] and a mix of PB2, PB1, PA and nucleoprotein (NP) expression plasmids (CA/04 or mutated) in quantities of 1, 1, 1, and 2 μg, respectively. The plasmid pGL4.75(*hRluc*/CMV), which expresses Renilla luciferase (Promega, Madison, WI, USA), was used as an internal control for a dual-luciferase assay. As a negative control, 293T cells were transfected with the same plasmids, with the exception of the NP expression plasmid. After 24 h of incubation at 37°C cell extracts were harvested and lysed, and luciferase levels were assayed with a dual-luciferase assay system (Promega) and a Synergy 2 multimode microplate reader (BioTek Instruments, Winooski, VT, USA). Experiments were performed in triplicate.

### Measurement of IFN gene expression

Quantification of changes in IFN gene expression was carried out by quantitative real-time polymerase-chain reaction (qPCR) measurement of individual IFN genes (*i*.*e*., *IFNB1*, *IFNL1*, and *IFNL2/3*). Total cellular RNA was isolated from virus-infected Calu-3 cells (MOI = 1) using RNeasy Minikit (Qiagen, Germantown, MD, USA). The RNA samples were then treated with DNase, and 1 μg of each purified RNA sample was then reverse-transcribed to cDNA with Quantiscript reverse transcriptase (Qiagen). The cDNAs were mixed with RT^2^ SYBR^®^ green qPCR Mastermix (Qiagen), and qPCR analyses were performed using the ViiA^™^ 7 instrument (Applied Biosystems, Waltham, MA, USA). *IFNB1*, *IFNL1*, and *IFNL2/3* gene copy numbers were assayed using Taqman gene expression assay primer/probe sets and master mix (Life Technologies, Carlsbad, CA, USA) and ViiA^™^ 7 software v.1.2.2 (Applied Biosystems). The values were determined by comparison to standard curves for each gene. Graphing and statistical analysis of the qPCR results were performed using Prism 6.0 (GraphPad Software). Values are the means of three independent determinations.

### ELISA

The secreted levels of IFN-λ1 and IFN-λ2/3 from Calu-3 cell culture supernatants were analyzed using ELISA kits supplied by BioLegend and RayBiotech, Inc., respectively. The IFNs levels from cell culture supernatants collected after infection with H1N1 viruses were analyzed in one experiment.

### Western blot

The levels of tyrosine-phosphorylated STAT1, RIG-I, IFN regulatory factor 3 (IRF3), and phospho-IRF3 were measured by Western blot analysis as described previously [22,46]. Whole cell lysates were prepared from virus-infected Calu-3 cultures (MOI = 1) at the indicated time points. Proteins were resolved by electrophoresis on 8% polyacrylamide Tris-Glycine gels (Invitrogen, Carlsbad, CA, USA), and then transferred to polyvinylidene difluoride (PVDF) membranes. The levels of tyrosine-phosphorylated STAT1, RIG-I, IRF3, and phospho-IRF3 were then measured by immunoblotting with mouse monoclonal anti-phospho-Y^701^-STAT1 Ab, rabbit monoclonal anti-RIG-I Ab, rabbit monoclonal anti-IRF3 Ab, and rabbit monoclonal phospho-IRF3, respectively (Cell Signaling Technology, Beverly, MA, USA). The levels of viral PB1 and M1 proteins in H1N1 viruses carrying silent PB1 and M1 mutations were analyzed by western blot with rabbit anti-PB1 (ThermoFisher Scientific, Rockford, IL, USA) and mouse monoclonal anti-M1 Abs (Abcam, Cambridge, MA, USA), respectively.

### Statistical analysis

The EC_50_ values, virus yield, plaque size and number, binding to sialyl receptors, NA enzyme kinetic parameters (*K*_*m*_ and *V*_max_), polymerase activities of ribonucleoprotein (RNP) complexes, and IFN gene/protein expression values induced by the wild-type and mutant H1N1 influenza viruses were compared by unpaired *t*-test or analysis of variance (ANOVA). Probability values ≤ 0.05 indicate statistically significant differences.

## Results

### Generation of IFN-λ1-resistant H1N1 mutants in Calu-3 cells

**Fig 1:**
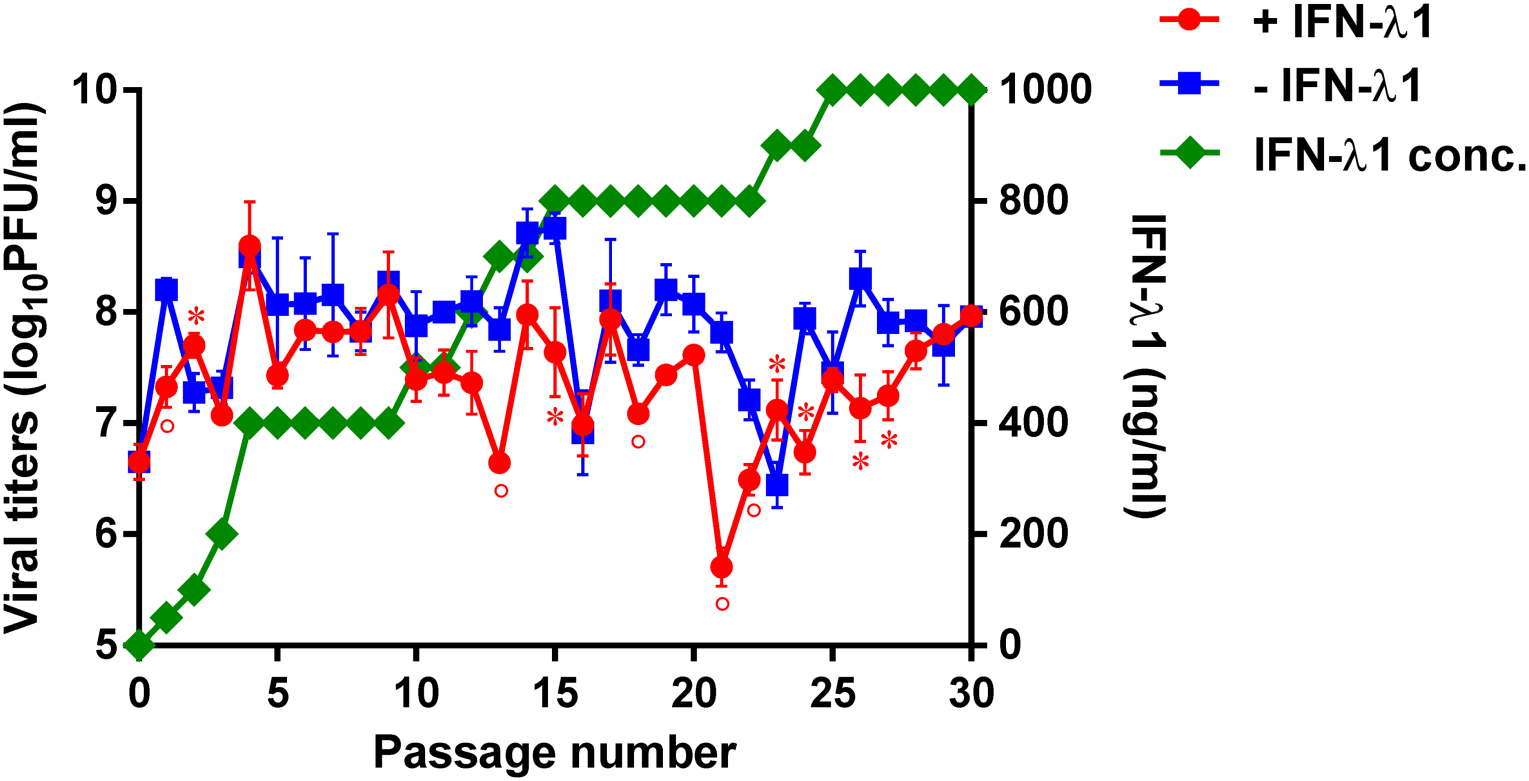
Generation of influenza A viruses with decreased sensitivity to IFN-λ1. A/California/04/09 virus was passaged in Calu-3 cells in the presence (red line) or absence (blue line) of increasing concentrations of IFN-λ1 (green line). *P < 0.05, °*P* < 0.01, compared to the values for the CA/04^–IFN-λ1^ virus.

We first measured the sensitivity of influenza A/California/04/09 (H1N1) virus to treatment with recombinant IFN-λ1. We evaluated the antiviral activity of this agent by cell ELISA, an assay that measures the level of inhibition of cell-associated virus yield in Calu-3 cells. A/California/04/09 virus was sensitive to inhibition by IFN-λ1: mean EC_50_ = 45.0 ± 8.4 ng/ml. However, IFN-λ1 did not fully protect Calu-3 cells from influenza H1N1 virus infection as it was observed previously [36]. This EC_50_ value was selected as the initial passage concentration of IFN-λ1 to obtain drug-resistant H1N1 mutants (Fig 1). We then serially passaged the A/California/04/09 strain 30 times in Calu-3 cells in the presence of increasing concentrations of IFN-λ1 to provide an opportunity for selection of an IFN-λ1-resistant viral phenotype. Virus yields were measured by plaque assay using MDCK cells after each passage. To monitor the emergence of any adaptive amino acid changes due to repeated passage in Calu-3 cells, we also passaged the parental virus in parallel without any selective pressure (*i*.*e*., no IFN-λ1).

Analysis of the A/California/04/09 virus following culture in the presence of increasing concentrations of IFN-λ1 showed the largest decrease in plaque number at passage 21 (5.7 ± 0.2 log_10_PFU/ml), and a mixed viral population was detected by plaque morphology analysis of culture supernatants at passage 15 (data not shown). Notably, the parental virus remained sensitive to inhibition by IFN-λ1 by cell ELISA after 15 passages (data not shown). Passage of the A/California/04/09 virus in the absence of IFN-λ1 caused the greatest reduction in plaque number at passage 23 (6.5 ± 0.2 log_10_PFU/ml, data not shown).

### Interferon sensitivity and growth characteristics of selected H1N1 variants

**Fig 2:**
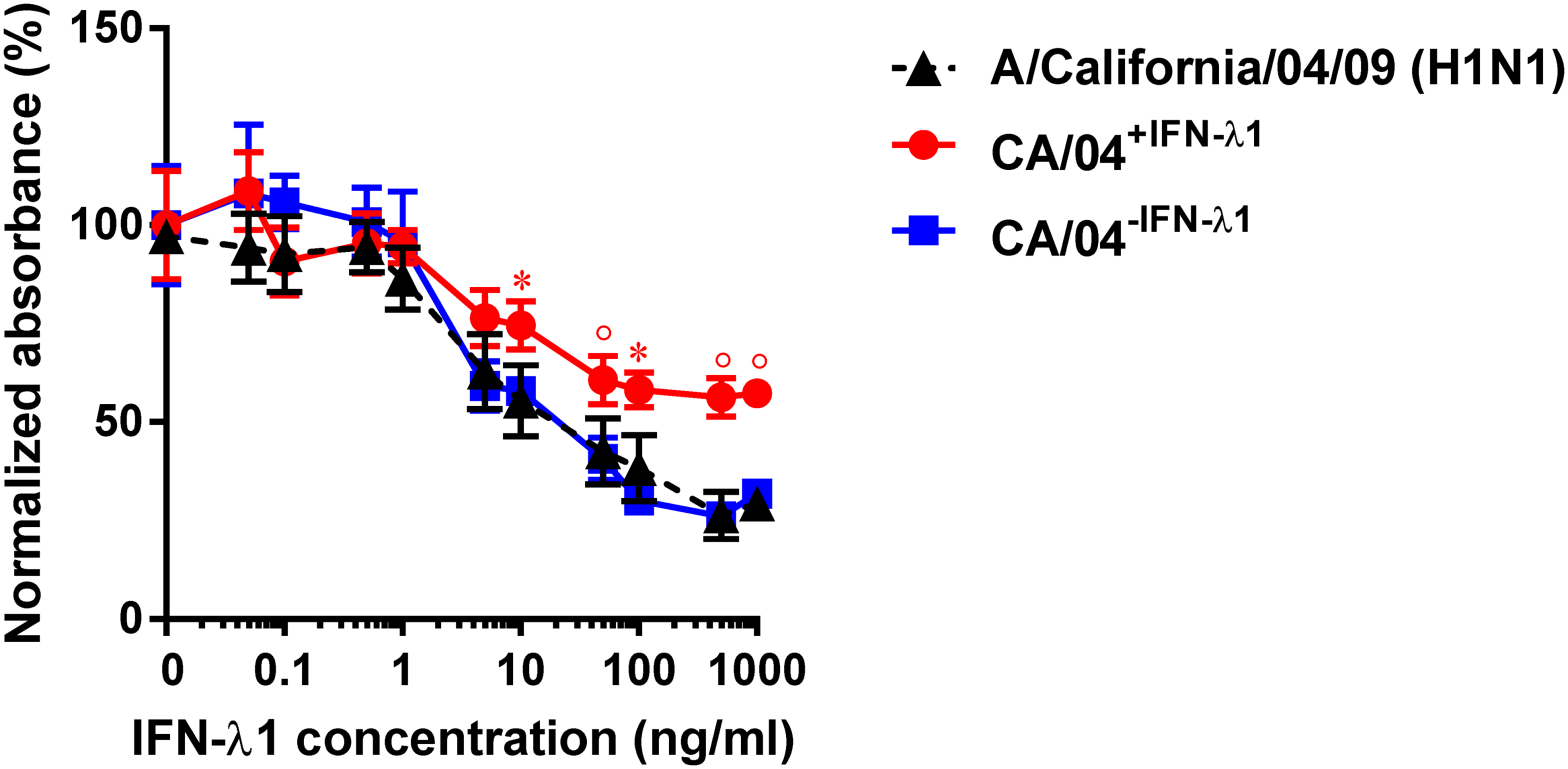
Antiviral activity of IFN-λ1 against the CA/04^+IFN-λ1^ and CA/04^–IFN-λ1^ viruses as determined by cell ELISA. *P < 0.05; °*P* < 0.01, compared to the values for the wild-type virus.

After 30 serial passages in the presence or absence of IFN-λ1, we prepared stocks of two selected H1N1 variants: CA/04^+IFN-λ1^ and CA/04^−IFN-λ1^ by plaque purification in MDCK cells. We then tested their sensitivity to the antiviral activity of IFN-λ1 by ELISA and by virus yield reduction assay. As shown in Fig 2, sensitivity of the CA/04^−IFN-λ1^ variant to IFN-λ1 was not significantly different from the wild-type virus (EC_50_ = 37.1 ± 1.9 ng/ml). In contrast, the CA/04^+IFN-λ1^ variant exhibited significantly reduced sensitivity to IFN-λ1 compared to the wild-type A/California/04/09 virus (EC_50_ ˃ 1000 ng/ml). The EC_50_ values determined by cell ELISA and by virus reduction assay (data not shown) were comparable. These findings indicated that the CA/04^+IFN-λ1^ variant may have acquired one or more mutation(s) that decreased its sensitivity to the antiviral activity of IFN-λ1.

**Fig 3:**
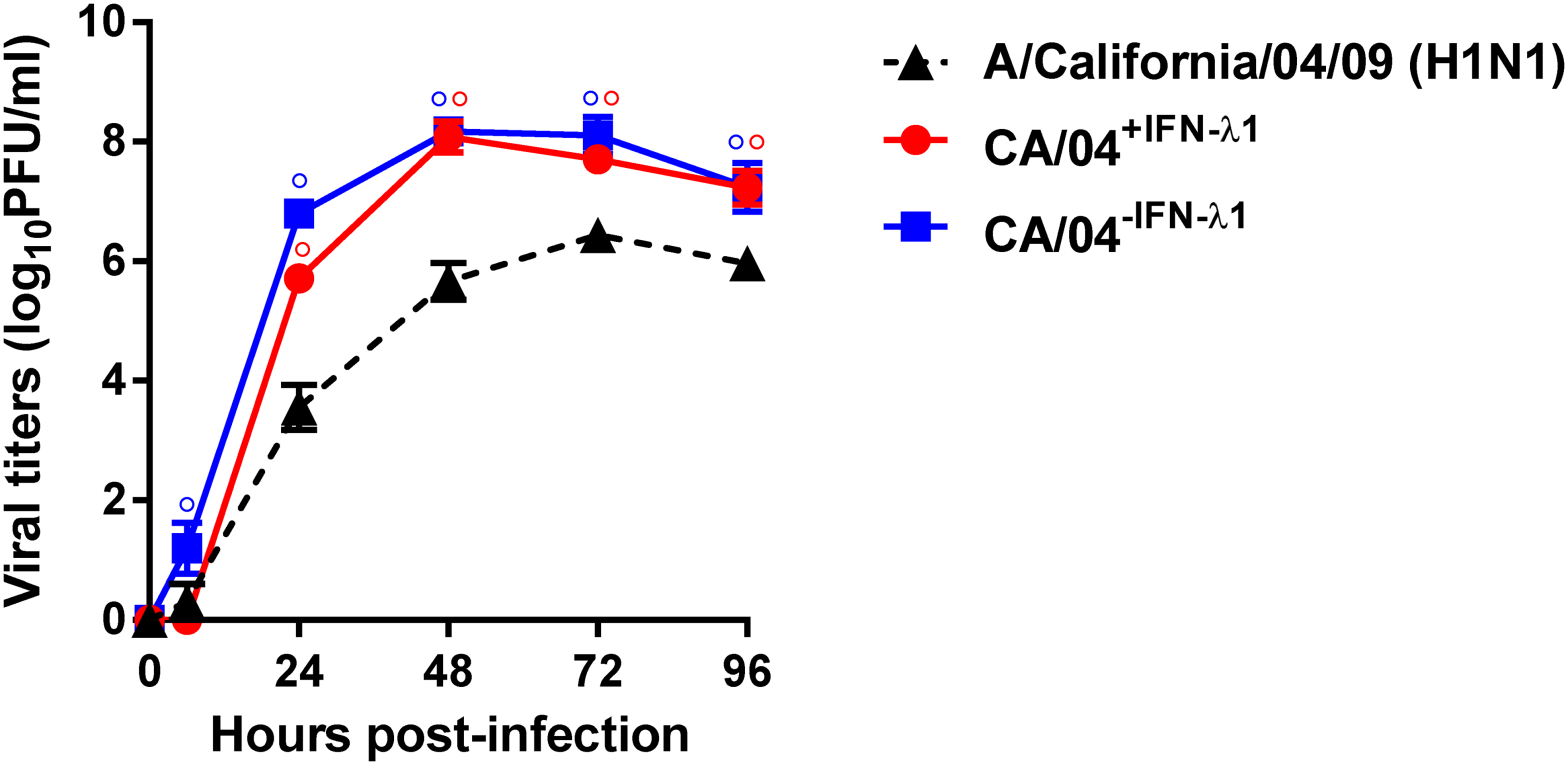
Replication of the A/California/04/09, CA/04^+IFN-λ1^ and CA/04^–IFN-λ1^ viruses in Calu-3 cells. The results are expressed as log_10_PFU/ml from three independent experiments. °*P* < 0.01, compared to the values for the wild-type virus.

We next examined growth of the wild-type, CA/04^+IFN-λ1^ and CA/04^−IFN-λ1^ viruses in MDCK cells. Both of the passaged variants grew to significantly higher titers than the parental strain, and they formed larger and more homogeneous plaques than the wild-type virus (*P* < 0.01, Table 1). To further evaluate the replicative ability of the selected H1N1 variants, we assayed their virus yields in comparison to those of the parental strain after multiple replication cycles in Calu-3 cells. As shown in Fig 3, the CA/04^+IFN-λ1^ virus grew faster and to significantly higher titers than the wild-type virus at 24, 48, 72 and 96 h post-infection (*P* < 0.01). The yields of the CA/04^−IFN-λ1^ variant were also significantly higher than the parental virus at all time points (↑~1.9 log, *P* < 0.01). These findings indicate that sequential passage of the parental H1N1 influenza virus in Calu-3 cells in the presence or absence of IFN-λ1 promoted selection of variants with enhanced growth properties.

**Table 1:** Growth characteristics of wild-type and two passaged H1N1 influenza viruses.

Viruses	Virus yield (log_10_PFU/ml)^a^	Plaque size (mm)^b^
A/California/04/09	6.7 ± 0.2	0.2 ± 0.1
CA/04^+IFN-λ1^	7.7 ± 0.2°	1.1 ± 0.4°
CA/04^−IFN-λ1^	7.9 ± 0.3°	1.1 ± 0.3°

^*a*^ Values represent the mean log_10_PFU/ml ± standard deviations from three independent determinations. The number of PFU in MDCK cells was measured by plaque assay after incubation at 37°C for 3 days with 10-fold serial dilutions of virus [38].

^*b*^ Values represent the mean plaque diameter (mm ± standard deviations) as measured by use of a Finescale comparator.

°*P* < 0.01, compared to the values for the wild-type virus by one-way ANOVA.

### Sequence analysis of selected H1N1 variants

To identify potential amino acid changes in the H1N1 influenza virus after culture in the presence or absence of IFN-λ1, we sequenced the complete genomes of our two selected variants (CA/04^+IFN-λ1^ and CA/04^−IFN-λ1^) at the end of the passaging protocol (Table 2). Sequence analysis mapped the acquired mutations to HA1, NA, M1, M2, and two polymerase proteins (PB1 and PA). A total of 4 nucleotide changes and 7 amino acid substitutions affecting six viral proteins were identified. We found that passage of the A/California/04/09 strain in the presence of IFN-λ1 resulted in development of two mutations in HA1 (G155E and S183P, H1 numbering used throughout the text), two mutations in NA (S79L and K331N), one mutation in M2 (E70K), and a single nucleotide mutation *T2136C* (italic font indicates nucleotide mutation representing silent mutation here and throughout the text) or *A183G* in PB1 or M1, respectively. Sequence analysis of the genome of the CA/04^−IFN-λ1^ virus revealed three mutations in HA1 (G155E, S183P, and M257I), one mutation in M2 (E70K), two nucleotide mutations in PB1 (*C975T* and *T2136C*), and one nucleotide mutation (*C1149T*) and one amino acid substitution (V14I) in the PA protein (Table 2). Because several identical mutations were generated in the HA1, M2, and PB1 genes when H1N1 virus was cultured with or without selective pressure, we conclude that the *T2136C* mutation in PB1, E70K mutation in M2, and the G155E and S183P mutations in HA1 are necessary for productive replication of this virus in Calu-3 cells, but are not required for decreased sensitivity to IFN-λ1-mediated antiviral activity.

**Table 2:** Nucleotide and amino acid substitutions identified in selected H1N1 influenza viruses.

Proteins	CA/04^+IFN-λ1^	CA/04^−IFN-λ1^
PB1	*T2136C*^b^	*C975T; T2136C*
PA	–	V14I, *C1149T*
HA1^a^	G155E^b^, S183P^b^	G155E, S183P, M257I
NA^a^	S79L^c^, K331N^c^	–
M1	*A183G*^b^	–
M2	E70K^b^	E70K

^*a*^ H1 and N1 numbering.

^*b*^ Mutations occurred before passage 15.

^*c*^ Mutations occurred after passage 15.

Italic indicates nucleotide changes.

Note: Silent mutations in PB1 protein did not affect protein expression (S1 Fig).

To determine if the amino acid changes identified in our CA/04^+IFN-λ1^ and CA/04^−IFN-λ1^ variants might also occur in other pandemic 2009 H1N1 isolates, we analyzed ~16,600 H1N1 genomic sequences deposited in the Influenza Research Database (data were obtained from the National Institute of Allergy and Infectious Diseases (NIAID) database: www.fludb.org). Three of the amino acid substitutions that we identified in our study, V14I in PA and G155E and S183P in HA1, were also present in 3.3% of the related H1N1 strains in the Influenza Research Database. The remaining eight mutations (*C975T*, *T2136C* in PB1, *C1149T* in PA, M257I in HA1, S79L, K331N in NA, *A183G* in M1, and E70K in M2) were found in <0.6% of contemporary H1N1 isolates.

### Effects of HA1, PA, and M mutations on receptor specificity, viral polymerase activity, and viral replication

**Fig 4:**
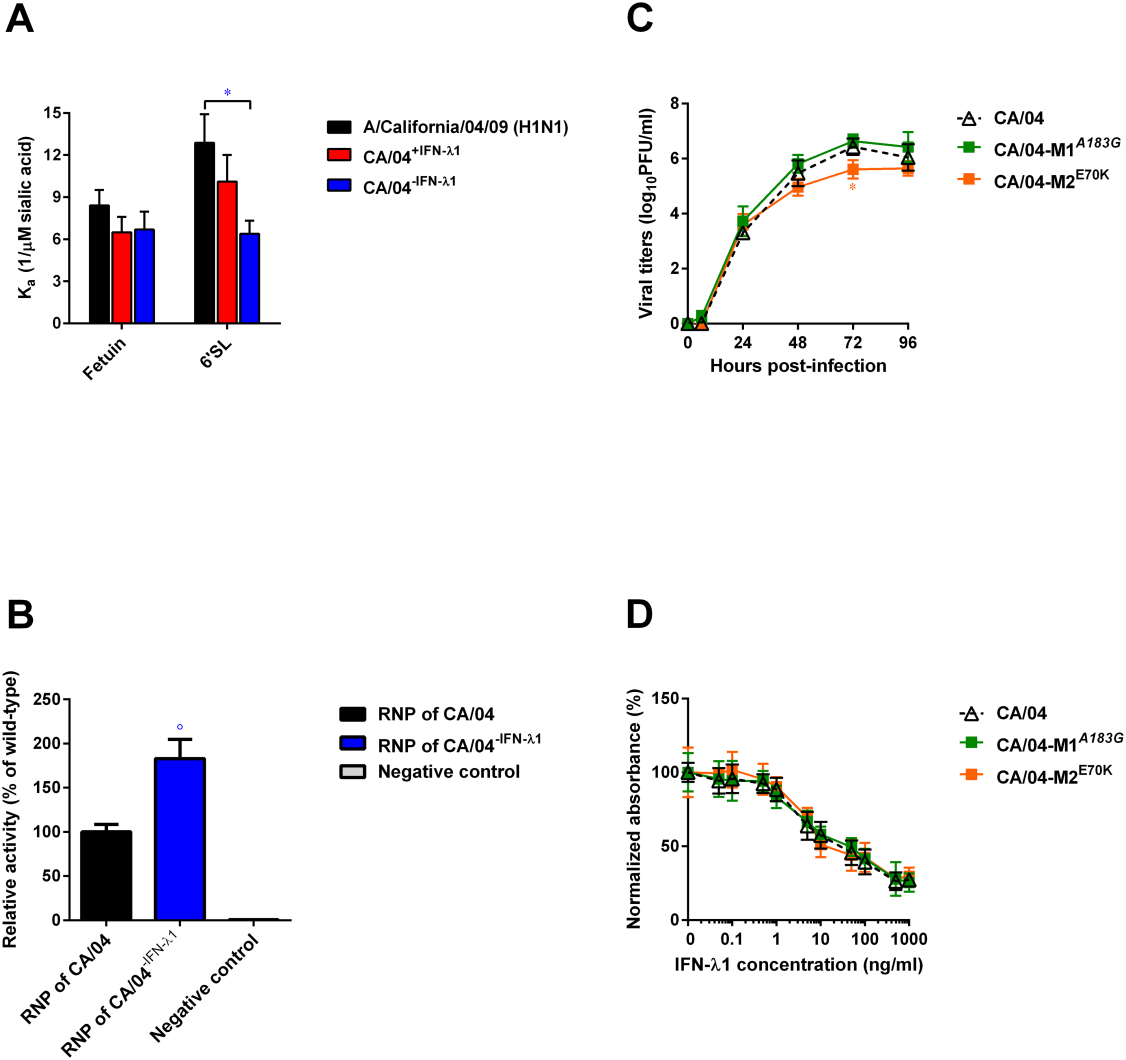
**(A) Receptor specificity of A/California/04/09, CA/04**^**+IFN-λ1**^, **and CA/04**^**–IFN-λ1**^ **viruses.** *P < 0.05, compared to the values for the wild-type virus. **(B) Polymerase activity of RNP complexes of wild-type and CA/04**^**–IFN-λ1**^ **containing PA V14I mutation.** The values represent the means ± standard deviations of activity of each RNP complex relative to that of CA/04 virus. °*P* < 0.01, compared to the values for the CA/04 virus. **(C) Replication efficiency of CA/04, CA/04-M1**^***A183G***^, **and CA/04-M2**^**E70K**^ **viruses in Calu-3 cells.** Representative results expressed as log_10_PFU/ml from three independent experiments are shown. *P < 0.05, compared to the values for the CA/04 virus. **(D) Antiviral activity of IFN-λ1 against CA/04, CA/04-M1**^***A183G***^, **and CA/04-M2**^**E70K**^ **viruses as measured by cell ELISA.**

To determine if the HA1 mutations that we identified affect the HA affinity for sialyl receptors, we examined the receptor specificity of two selected variants, CA/04^+IFN-λ1^ and CA/04^−IFN-λ1^, in comparison to the wild-type virus. The binding level to the “avian-type” receptor (3′SL/N) was negligible (data not shown). Based on the *K*_a_ values (Fig 4A), both of our H1N1 variants demonstrated identical binding to fetuin. However, the CA/04^−IFN-λ1^ virus exhibited a significantly lower affinity toward the 6′SL polymer compared to the wild-type virus (↓∼2-fold; *P* < 0.05).

To determine if the observed amino acid substitution V14I in the PA protein of the CA/04^−IFN-λ1^ variant altered its viral transcription activity, we analyzed reconstituted RNP complexes in 293T cells using a luciferase mini-genome reporter assay (Fig 4B). We found that the single point mutation, PA V14I, significantly increased polymerase activity of the A/California/04/09 polymerase complex as much as 180% (*P* < 0.01).

To examine the potential impact of mutations in the M gene, we used reverse-genetic technique. We introduced the *A183G* nucleotide change and the E70K amino acid mutation into the background of the wild-type CA/04 virus to generate the recombinant viral constructs, CA/04-M1^*A183G*^ and CA/04-M2^E70K^. Our data showed that *A183G* change slightly increased M1 protein expression level (S1 Fig). We then determined whether either of these changes affected growth rates of the recombinant H1N1 viruses. As shown in Fig 4C, levels of the CA/04-M2^E70K^ virus were significantly lower than the CA/04 virus only at 72 h post-infection (*P* < 0.05). We next measured sensitivity of our H1N1 recombinant viruses to the antiviral activity of IFN-λ1 by cell ELISA. Both of the mutant viruses, CA/04-M1^*A183G*^ and CA/04-M2^E70K^, exhibited sensitivity to IFN-λ1 that was equivalent to the wild-type CA/04 strain in Calu-3 cells (Fig 4D).

### Effects of NA amino acid substitutions on viral growth, neuraminidase activity and sensitivity to IFN-λ1

**Fig 5:**
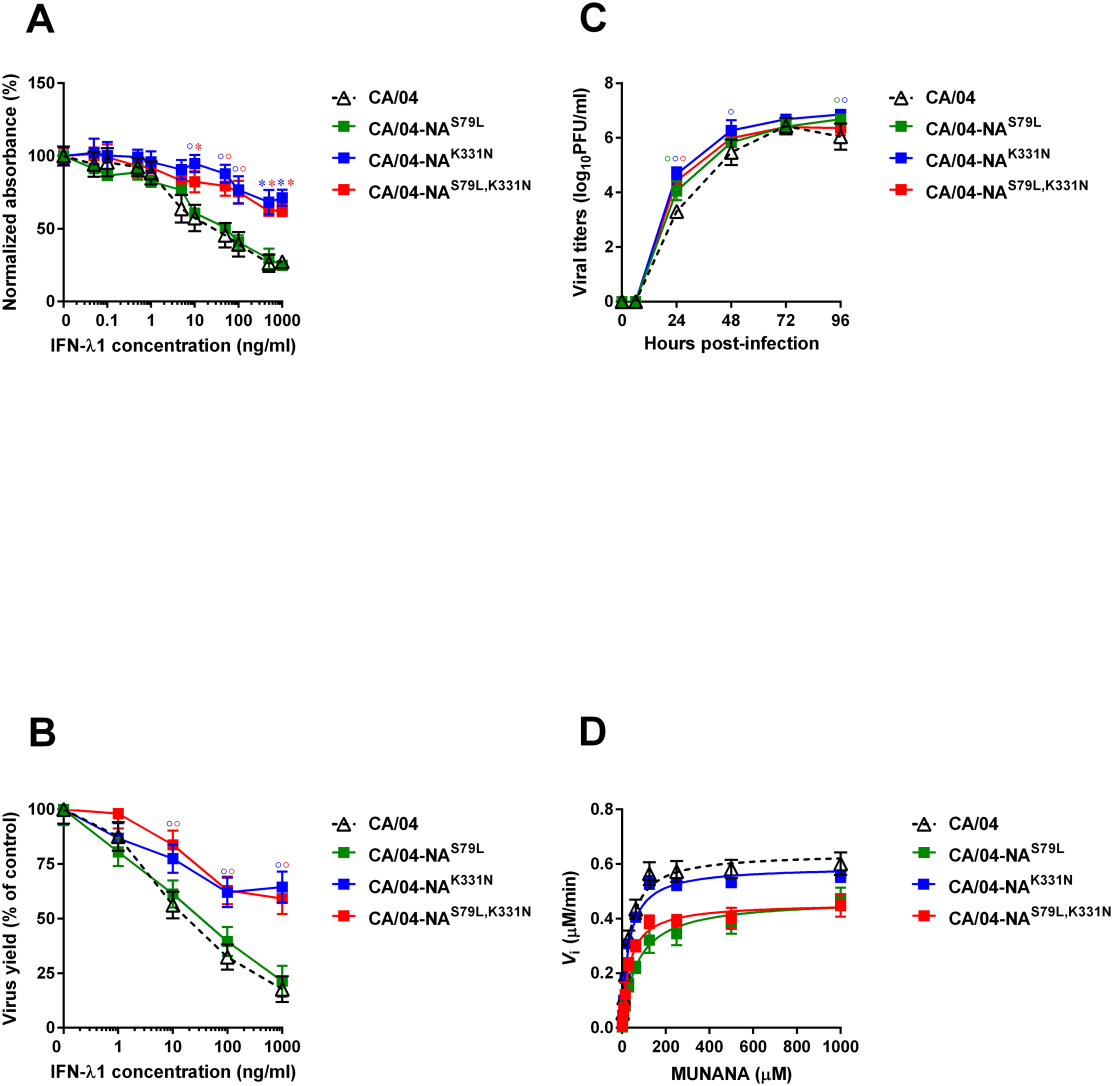
**Antiviral effect of IFN-λ1 in Calu-3 cells as measured by cell ELISA (A) and by virus reduction assay (B).** *P < 0.05, °*P* < 0.01, compared to the values for the parental CA/04 virus. **(C) Replication efficiencies of the CA/04, CA/04-NA**^**S79L**^, **CA/04-NA**^**K331N**^, **and CA/04-NA**^**S79L,K331N**^ **viruses in Calu-3 cells.** *P < 0.05, °*P* < 0.01, compared to the values for the CA/04 virus. **(D) NA enzyme kinetics of CA/04, CA/04-NA**^**S79L**^, **CA/04-NA**^**K331N**^, **and CA/04-NA**^**S79L,K331N**^ **viruses.** Substrate conversion velocity (*V*_i_) of NA was measured as a function of substrate concentration.

To determine if the NA mutations found in the CA/04^+IFN-λ1^ virus affected viral responsiveness to IFN-λ1, we introduced the S79L and K331N mutations into the background of the parental CA/04 virus to generate three recombinant viral constructs: CA/04-NA^S79L^, CA/04-NA^K331N^, and CA/04-NA^S79L,K331N^. These recombinant viruses were successfully rescued, and sequence analysis of these viruses showed that the incorporated NA mutations were stably maintained in the CA/04 virus background. No additional HA or NA mutations were found (data not shown). We first measured the sensitivity of these three recombinant viruses (CA/04-NA^S79L^, CA/04-NA^K331N^, and CA/04-NA^S79L,K331N^) to the antiviral activity of IFN-λ1 by cell ELISA using Calu-3 cells as target cells (Fig 5A). The mean EC_50_ value for the CA/04-NA^S79L^ virus was 53.7 ± 6.7 ng/ml, which was very similar to that of the wild-type CA/04 strain. In contrast, the two other recombinant viruses, CA/04-NA^K331N^ and CA/04-NA^S79L,K331N^, were significantly more resistant to IFN-λ1 than the parental CA/04 virus (EC_50_ ˃ 1000 ng/ml). We also observed that the EC_50_ values determined by cell ELISA and by virus reduction assay (Fig 5B) were comparable.

We next examined replication of the CA/04-NA^S79L^, CA/04-NA^K331N^, and CA/04-NA^S79L,K331N^ variants in MDCK cells. The CA/04-NA^K331N^ and CA/04-NA^S79L,K331N^ viruses grew to significantly higher titers than the wild-type virus in MDCK cells (Table 3, *P* < 0.05). We also noted that the plaque sizes of all three mutant viruses were significantly increased (↑~4.1-fold) compared to those of the parental CA/04 virus (Table 3, *P* < 0.05). To further evaluate the replication ability of our recombinant H1N1 viruses carrying the different NA mutations, we assayed their viral yields in comparison to the wild-type CA/04 strain after several replication cycles in Calu-3 cells. As shown in Fig 5C, the CA/04-NA^S79L,K331N^ virus grew to significantly higher titers than the wild-type CA/04 virus at 24 h post infection (↑∼0.8 logs, *P* < 0.01). Furthermore, the yields of the CA/04-NA^S79L^ and CA/04-NA^K331N^ mutants were also higher than that of the wild-type virus at 24 and 96 h post-infection time points in Calu-3 cells (↑∼0.9 logs, *P* < 0.01).

**Table 3:** Growth characteristics and NA enzymatic properties of recombinant H1N1 influenza viruses.

Viruses	Virus yield (log_10_PFU/ml)^a^	Plaque size (mm)^b^	*V*_max_ (μM/min)^c^	*K*_m_ (μM)^d^
CA/04	6.7 ± 0.2	0.2 ± 0.1	0.64 ± 0.02	30.75 ± 3.59
CA/04-NA^S79L^	6.6 ± 0.2	0.6 ± 0.1*	0.47 ± 0.02*	69.59 ± 9.43*
CA/04-NA^K331N^	7.2 ± 0.2*	1.1 ± 0.2*	0.59 ± 0.02	30.42 ± 4.09
CA/04-NA^S79L,K331N^	7.4 ± 0.3*	1.0 ± 0.2*	0.46 ± 0.02*	34.56 ± 4.33

^*a*^ Values represent the mean log_10_PFU/ml ± standard deviations from three independent determinations. The number of PFU in MDCK cells was measured by plaque assay after incubation at 37°C for 3 days with 10-fold serial dilutions of virus [38].

^*b*^ Values represent the mean plaque diameter (mm ± standard deviations) as measured by use of a Finescale comparator.

^*c*^ The *V*_max_ was calculated using a nonlinear regression of the curve according to the Michaelis-Menten equation.

^*d*^ The *K*_m_ represents the Michaelis-Menten constant (μM) at which the reaction rate is half of *V*_max_. The enzyme kinetic data were fit to the Michaelis-Menten equation using GraphPad Prism, version 6.0. Values are the means ± standard deviations from three independent determinations.

*P < 0.05, compared to the values for the wild-type virus by one-way ANOVA.

To evaluate the impact of the S79L and K331N mutations on NA enzyme activity, we determined the NA enzyme K_*m*_ and *V*_max_ values for the CA/04, CA/04-NA^S79L^, CA/04-NA^K331N^, and CA/04-NA^S79L,K331N^ viruses (Fig 5D and Table 3). We observed that although the K331N mutation had no effect on the K_*m*_ and *V*_max_ values, NA protein containing the S79L mutation exhibited significantly higher affinity for the substrate (mean *K*_m_, ↑2.3-fold) than the wild-type virus NA (Table 3, *P* < 0.05). Furthermore, the S79L mutation correlated with significantly decreased NA enzyme activity (*V*_max_ ratio relative to the wild-type virus = 0.7; Fig 5D and Table 3).

### Effects of NA amino acid substitutions on innate immune response

**Fig 6:**
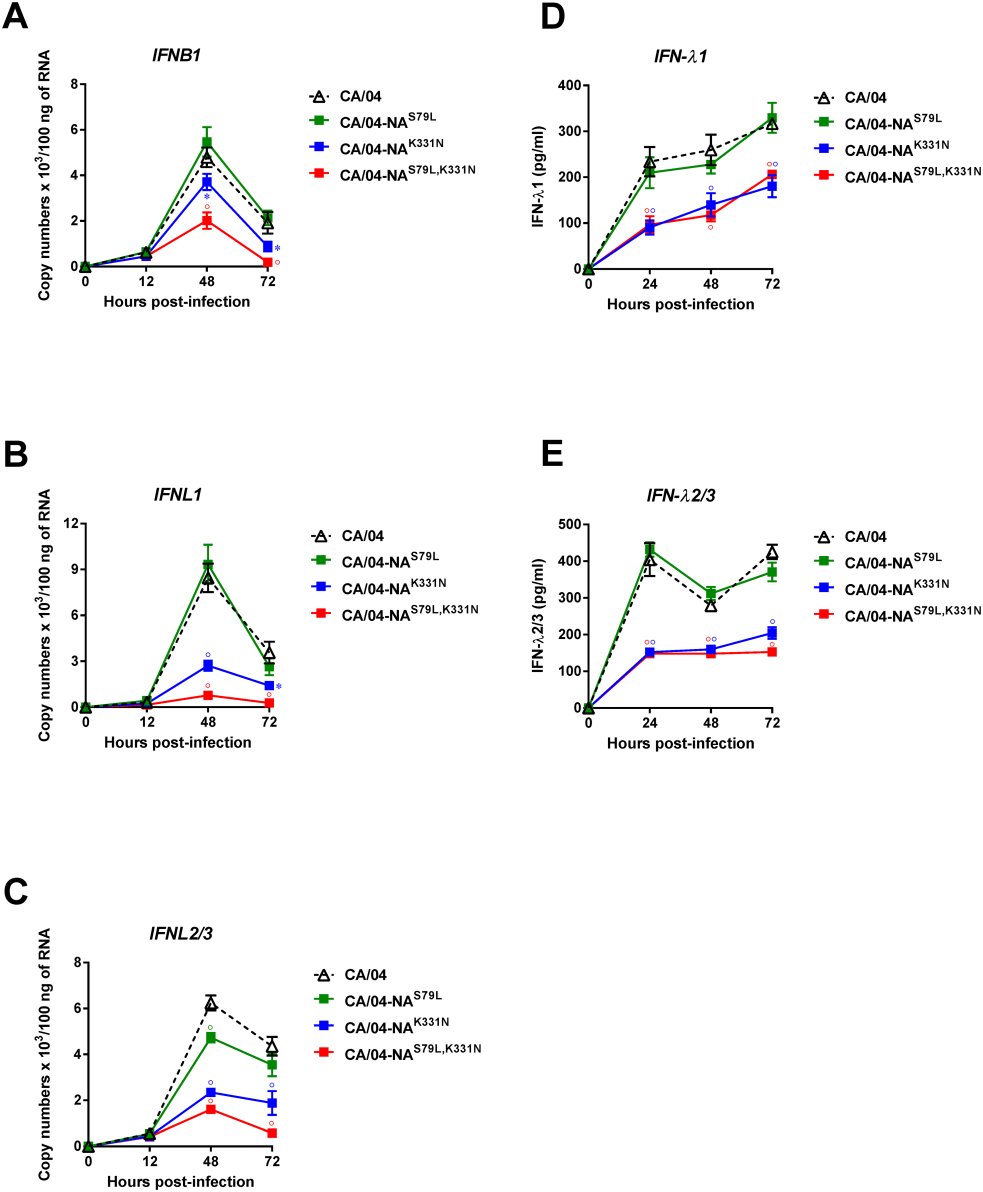
**(A, B, C) Influenza-induced IFN gene expression levels in Calu-3 cells.** Cells were infected with the indicated recombinant viruses (MOI = 1) and the levels of IFNs were quantified by qPCR at 12, 48, and 72 hpi. Values were determined by comparison to standard curves for each gene and the results are expressed as RNA copy numbers. *P < 0.05; °*P* < 0.01, compared to the values for the CA/04 virus. **(D, E) IFN-λ1 and –λ2/3 protein production in Calu-3 cells.** Cells were infected with viruses at a MOI of 5. Supernatants were collected at 24, 48, and 72 hpi, and the levels of secreted IFN-λ1 protein were determined by ELISA. °*P* < 0.01, compared to the values for the CA/04 virus.

We compared the ability of our recombinant H1N1 viruses containing the NA mutations to induce IFN gene expression (*IFNB1*, *IFNL1*, and *IFNL2/3*) in Calu-3 cells. As shown in Fig 6A–6C, the CA/04-NA^S79L^ virus induced similar levels of IFN gene expression as those induced by the parental virus. However, both the CA/04-NA^K331N^ and CA/04-NA^S79L,K331N^ viruses induced significantly lower levels of *IFNB1*, *IFNL1*, and *IFNL2/3* gene expression at 48 and 72 h post-infection (*P* < 0.05). We next measured IFN-λ1 and IFN-λ2/3 protein levels in Calu-3 cells after infection with our mutant viruses by ELISA (Fig 6D and 6E). The levels of IFN-λ1 and IFN-λ2/3 production correlated well with the levels of *IFNL1* and *IFNL2/3* gene expression. The IFN-λs protein levels at individual time points were significantly lower after infection with CA/04-NA^K331N^ and CA/04-NA^S79L,K331N^ compared to infection with the parental CA/04 strain or the CA/04-NA^S79L^ viruses (*P* < 0.01).

**Fig 7:**
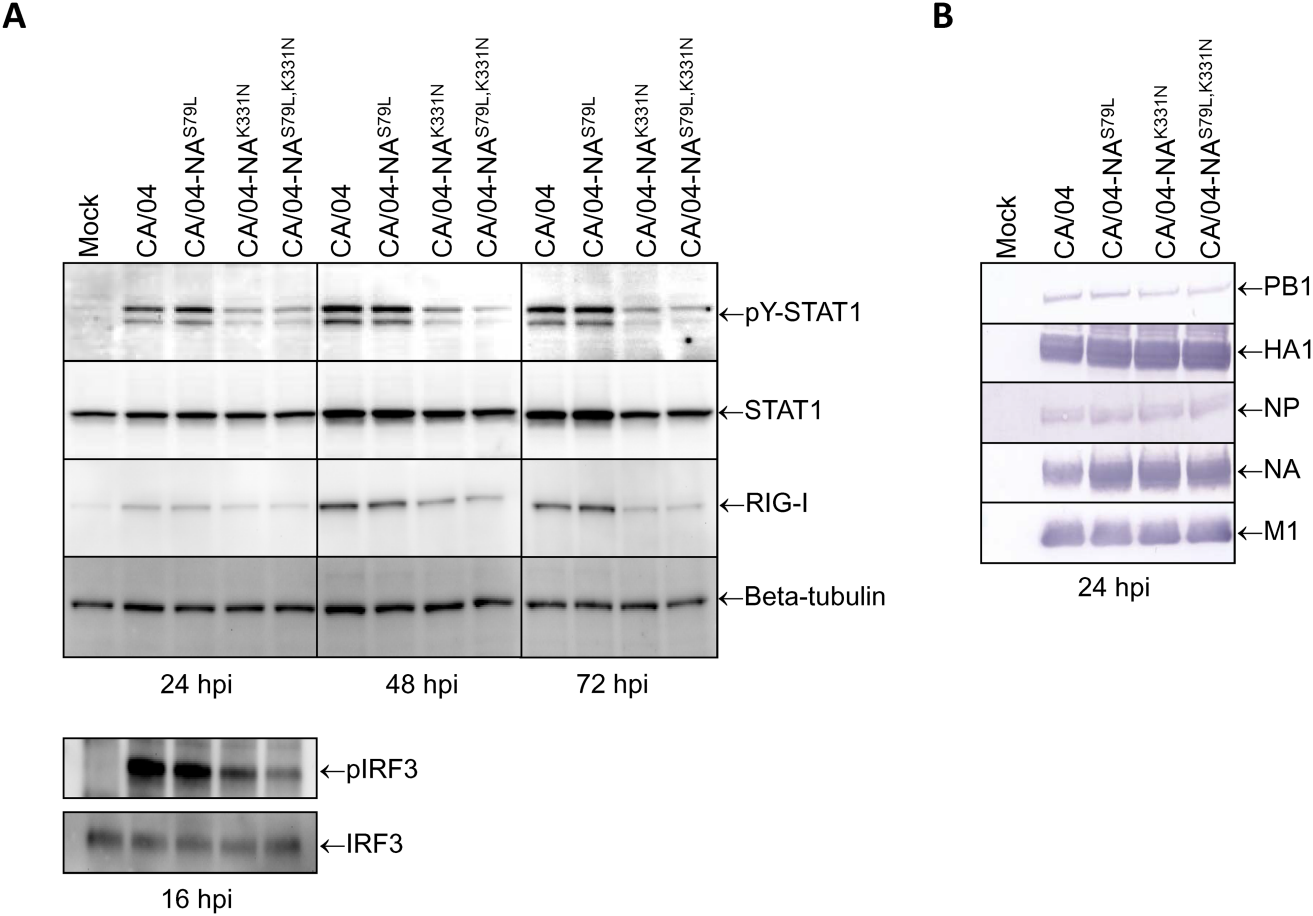
**(A) Comparison of the levels of tyrosine-phosphorylated STAT1, RIG-I, and IRF3 phosphorylation in influenza-infected Calu-3 cells.** Cells were infected with the mutant viruses (MOI = 1) and incubated for 16, 24, 48 or 72 h. At the specified time points, whole cell lysates were prepared and the levels of STAT1 activation, RIG-I expression, and IRF3 phosphorylation were measured by Western blot analysis. Total cellular STAT1 and beta-tubulin protein levels were analyzed to control for equal loading. hpi—hours post-infection. **(B) Comparison of the PB1, HA1, NA, NP, and M1 levels in influenza-infected Calu-3 cells.** Calu-3 cells were infected with the mutant viruses (MOI = 1) and incubated for 24 h. Whole cell lysates were prepared and the levels of viral proteins were measured by Western blot analysis. hpi—hours post-infection.

To determine if the reduced levels of IFN gene expression shown in Fig 6A–6C correlated with decreased STAT1 activity and/or reduced RIG-I protein levels, we infected Calu-3 cells with our mutant viruses (MOI = 1), and incubated the cultures for 24, 48 or 72 h. At the specified time points, whole cell lysates were prepared, and the levels of STAT1 activation and RIG-I expression were measured by Western blot analysis. As shown in Fig 7A, the levels of tyrosine-phosphorylated STAT1 and RIG-I were lower at 24, 48 and 72 h post-infection with either the CA/04-NA^K331N^ or CA/04-NA^S79L,K331N^ viruses compared to infection with the parental CA/04 strain or the CA/04-NA^S79L^ viruses.

Since activation of IRF3 is essential for induction of IFN gene expression, we determined if there is a difference in the ability of our viruses to induce activation of this transcription factor in Calu-3 cells. We found that phosphorylation of IRF3 was decreased in cells infected with the CA/04-NA^K331N^ and CA/04-NA^S79L,K331N^ variants compared to cells infected with the CA/04 or CA/04-NA^S79L^ viruses at 16 h post-infection (Fig 7A). We next examined if there is a difference in expression of the viral proteins after infection with CA/04, CA/04-NA^S79L^, CA/04-NA^K331N^, and CA/04-NA^S79L,K331N^ viruses. Our results showed higher expression of the NA and M1 proteins after infection with the mutant viruses compared to the parental strain at 24 h post-infection (Fig 7B). Taken together, our findings indicate that CA/04 virus containing either K331N or S79L, K331N mutations induces weaker phosphorylation of IRF3 and, as a consequence, lower levels of IFN gene expression. The decreased IFN gene expression levels correlated with decreased activation of STAT1 and reduced expression of RIG-I protein.

## Discussion

In the present study, we generated an IFN-λ1-resistant mutant of the A/California/04/09 (H1N1) virus by serially passaging the parental virus in the presence of increasing concentrations of IFN-λ1 for a total of 30 passages. The selective pressure mediated by IFN-λ1 induced acquisition of several viral genomic mutations. One of these mutations, K331N in the NA protein, correlated with weaker phosphorylation of IRF3 and decreased ability of the virus to induce expression of the IFN genes (*IFNB1*, *IFNL1*, *IFNL2/3*) and IFN proteins (IFN-λ1 and IFN-λ2/3) by target respiratory epithelial cells. The reduced IFN gene/protein expression levels also correlated with decreased activation of STAT1, suppressed expression of RIG-I, and reduced sensitivity to the antiviral activity of IFN-λ1.

In order to establish productive infection, influenza viruses have evolved several mechanisms to counteract the production and antiviral activity of interferons [47–53]. For example, influenza virus expresses the non-structural 1 (NS1) protein that can inhibit IFN induction using multiple strategies [54]. NS1 has been shown to bind dsRNA and mask viral RNA species from recognition by the host cells [55,56]. NS1 also interacts with retinoic acid-inducible gene I (RIG-I) and its co-activator, TRIM25, leading to impaired activation of the IRF3, ATF/c-Jun and NF-κB transcription factors that drive IFN-β gene expression [57–60]. In addition, NS1 can interact with PKR and inhibit its activation [61,62]. Additionally, recent reports describe a type I IFN antagonism function for the PB2, PA, and PB1-F2 proteins, which were found to interact with mitochondrial antiviral signaling protein (MAVS) and impair IFN-β production without affecting viral replication [47,63]. Furthermore, Xia et al. [64] demonstrated that the influenza surface glycoprotein, HA, is capable of promoting IFNAR1 ubiquitination and degradation. This results in a reduction of the levels of IFNAR1 expression and marked suppression of cellular responsiveness to type I IFNs.

We found that two amino acid changes in the NA protein, S79L and K331N, were selected in the A/California/04/09 virus after extended passaging in Calu-3 cells in the presence of IFN-λ1. The S79L mutation is located in the stem domain, and K331N is located near the active site of the NA protein. We observed that both of these NA mutations were viable and genetically stable on the H1N1 virus background. The S79L mutation significantly reduced the activity of the NA enzyme (*P* < 0.05); however, it did not compromise viral growth. Importantly, our results showed that the K331N but not the S79L NA mutation was associated with markedly increased resistance to IFN-λ1 (˃ 20-fold increase in the mean EC_50_) and correlated with reduced anti-viral responsiveness to IFN-λ1. We have not studied the impact of the K331N substitution on sensitivity to IFN-λ2 and -λ3 because of the initial resistance of the parental virus to these type III IFNs (data not shown).

Our results showed that the CA/04-NA^S79L,K331N^ variant exhibited reduced NA enzyme catalytic activity due to the S79L mutation, but this change was not associated with a reduction in viral replication in Calu-3 cells. Since IFN-λ1-induced selective pressure resulted in acquisition of both NA mutations and only one of these (K331N) was associated with the IFN-λ1-resistant phenotype, the decreased NA activity mediated by the S79L mutation may be important for H1N1 virus viability in human epithelial cells. This finding coincides with previously reported data showing that decreased NA activity is necessary for the adaptation of highly pathogenic H5N1 influenza viruses for growth in human airway epithelium [65]. Notably, A/California/04/09 (H1N1) exhibited weak NA enzymatic activity in catalyzing α2,6-linked glycans, which was necessary for efficient transmission between humans [66]. Additionally, in light of the interrelated functions of the HA and NA glycoproteins [67], the decrease in NA activity could involve acquisition of the compensating HA1 mutations (G155E and S183P) which were generated in the CA/04^+IFN-λ1^ variant that was selected in the presence of IFN-λ1. In this study we examined the phosphorylation status of IRF3 and the kinetic profile of IFN gene/protein expression by Calu-3 cells after infection with recombinant H1N1 viruses carrying the S79L and/or K331N mutations. We found that mutants carrying either the single amino acid change (K331N) or the double mutation (S79L and K331N) induced weaker antiviral responses than the parental CA/04 virus—as evidenced by reduced IRF3 phosphorylation, weak induction of the *IFNB1*, *IFNL1*, and *IFNL2/3* genes, and low expression of IFN-λ1 and IFN-λ2/3 proteins in Calu-3 cells. Moreover, infection with the CA/04-NA^K331N^ or CA/04-NA^S79L,K331N^ virus resulted in decreased activation of STAT1 and reduced expression of RIG-I compared to those induced by infection with the parental CA/04 virus or the CA/04-NA^S79L^ virus. It is unclear how influenza infection of Calu-3 cells pretreated with IFN-λ1 can select for a virus that induces less IRF3 phosphorylation and a consequent weaker production of IFN-λ1. Interestingly, previous study by Grimm et al. [68] demonstrated that influenza NA protein was capable of contributing to high virulence of a A/Puerto Rico/8/34 (H1N1) variant, which allowed the virus to escape mouse innate immune control due to the high speed of virus growth and early viral gene expression.

To monitor the potential emergence of amino acid changes associated with adaptation of the A/California/04/09 virus to growth in Calu-3 cells, we also passaged the parental virus in parallel in the absence of IFN-λ1. We identified three mutations in the HA1 protein (G155E, S183P, and M257I), one amino acid change in the PA protein (V14I), and one mutation in the M2 protein (E70K), that partially, and more likely independently, controlled adaptation of the pandemic 2009 isolate to growth in respiratory epithelial cells. Notably, two of the HA1 mutations that we identified in this study, G155E and S183P, were shown previously to be necessary for the enhanced virulence of the A/California/04/09 (H1N1) isolate in mice [69]. Further characterization of the receptor specificity of our CA/04^+IFN-λ1^ and CA/04^−IFN-λ1^ variants revealed that M257I HA1 substitution was associated with a significant decrease in binding to α2,6 sialic acid-linked receptor (6′SL, *P* < 0.05). It is likely that the observed HA1 changes located next to the HA receptor-binding pocket contribute to the increased ability of the parental virus to infect and spread in mammalian respiratory epithelium.

We found that mutations at four points in the RNP complex, namely PB1 *C975T*, PB1 *T2136C*, PA V14I, and PA *C1149T*, were involved in the adaptation of the pandemic H1N1 isolate to growth in Calu-3 cells. A single amino acid substitution (V14I) in the PA protein was sufficient to significantly enhance the transcription activity of the polymerase complex of the A/California/04/09 virus (*P* < 0.01). The V14I mutation occurred only in the CA/04^−IFN-λ1^ virus most likely due to the lack of additional IFN-λ1 selective pressure during sequential passaging. This mutation was likely necessary for optimal interaction of the viral polymerase with human host proteins and enabled the CA/04^−IFN-λ1^ virus to replicate efficiently in Calu-3 cells.

Passage of H1N1 virus in the presence or absence of IFN-λ1 resulted in acquisition of an M2 mutation, E70K, which was associated with decreased replication ability in Calu-3 cells at 72 h post-infection. However, since an amino acid change of the same residue (residue 70) occurred independently in both variants, CA/04^+IFN-λ1^ and CA/04^−IFN-λ1^, this indicates that this change is not a random mutation. We believe that this change reflects a specific character of variation, most likely linked to adaptation of this virus for growth in the human lower respiratory tract. Our results add to the growing body of mutational analysis data defining the structural and functional changes that govern adaptation of H1N1 viruses to human respiratory epithelial cells. Our findings demonstrate that multiple adaptive changes occurred when the A/California/04/09 (H1N1) virus was passaged for an extended time period in Calu-3 cells. These viral adaptations included optimizing receptor specificity, NA enzyme catalytic activity, and interaction of viral polymerase components with host cell factors. These changes provide a means by which the pandemic H1N1 virus can achieve an optimal competitive advantage in the human lower respiratory tract.

To our knowledge, this is the first study to evaluate the emergence of resistance of pandemic A/California/04/09 (H1N1) influenza virus to the anti-viral activity of a type III interferon. Future characterization of the genetic stability, infectivity, and pathogenicity of our IFN-λ1-resistant H1N1 virus in human primary lung epithelial cells or in an appropriate animal model should help to define its pathogenicity. Finally, the findings described in this report may aid in the development of more effective therapeutic strategies to prevent and treat influenza virus infection.

## Supporting information

S1 Fig PB1 and M1 protein expression levels in CA/04, CA/04^−IFN-λ1^, and CA/04-M1^*A183G*^ viruses. The levels of viral PB1 and M1 proteins in concentrated and purified H1N1 viruses carrying silent PB1 and M1 mutations were analyzed by western blot with rabbit anti-PB1 (ThermoFisher Scientific, Rockford, IL, USA) and mouse monoclonal anti-M1 Abs (Abcam, Cambridge, MA, USA), respectively.
(PPTX)
