# Supplementary figures and images for: Generation and characterization of interferon-lambda 1-resistant H1N1 influenza A viruses
Source: PLoS One. 2017 Jul 27;12(7):e0181999. doi: 10.1371/journal.pone.0181999 (PMC5531537; doi:10.1371/journal.pone.0181999)

## Slide 1
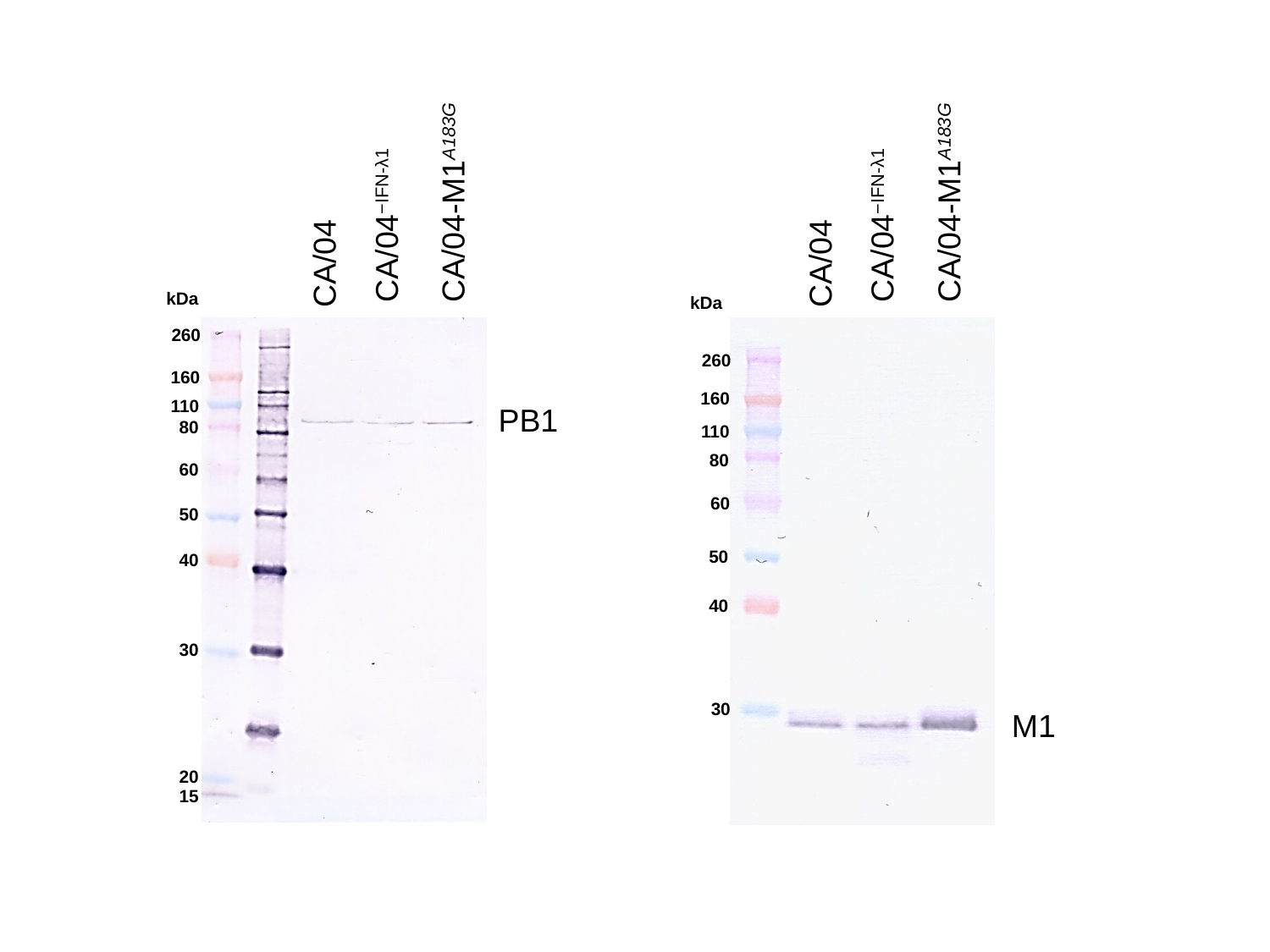

CA/04-M1A183G
CA/04-M1A183G
CA/04−IFN-λ1
CA/04−IFN-λ1
CA/04
CA/04
kDa
kDa
260
260
160
160
110
PB1
80
110
80
60
60
50
50
40
40
30
30
M1
20
15

Supplement: S1 Fig — The levels of viral PB1 and M1 proteins in concentrated and purified H1N1 viruses carrying silent PB1 and M1 mutations were analyzed by western blot with rabbit anti-PB1 (ThermoFisher Scientific, Rockford, IL, USA) and mouse monoclonal anti-M1 Abs (Abcam, Cambridge, MA, USA), respectively. (PPTX) [file pone.0181999.s001.pptx]
